# Supplementary material for: Sequence signatures of two public antibody clonotypes that bind SARS-CoV-2 receptor binding domain
Source: Nat Commun. 2021 Jun 21;12:3815. doi: 10.1038/s41467-021-24123-7 (PMC8217500; doi:10.1038/s41467-021-24123-7)
Supplement: Supplementary file 1 — Supplementary Information [file 41467_2021_24123_MOESM1_ESM.pdf]

Sequence signatures of two public antibody clonotypes that bind SARS-CoV-2 receptor binding domain

Supplementary Figures

a

CC12.1 (Rogers et al. 2020) – Clonotype 1

```
                    C  A  R  D  L  D  V  Y  G  L  D  V  W
                   TGTGCGAGAGATTTAGATGTCTACGGTTTGGACGTCTGG
Germline sequence: TGTGCGAGAGA   AGATG CTACGGTATGGACGTCTGG
                     IGHV3-53  IGHD5-24        IGHJ6
```

COV072_P3_HC_80-P1369 (Robbiani et al. 2020) – Clonotype 1

```
                    C  A  R  D  L  G  D  Y  G  M  D  V  W
                   TGTGCGAGAGATCTGGGGGACTACGGAATGGACGTCTGG
Germline sequence: TGTGCGAGAGA  TGGGGGACTACGGTATGGACGTCTGG
                     IGHV3-66  IGHD3-16       IGHJ6
```

COV2-2080 (Zost et al. 2020) – Clonotype 1

```
                    C  A  R  D  L  V  T  Y  G  L  D  V  W
                   TGTGCGAGAGATCTCGTGACTTACGGTTTGGACGTCTGG
Germline sequence: TGTGCGAGAGA    GTGACTTACGGTATGGACGTCTGG
                     IGHV3-66    IGHD2-21      IGHJ6
```

1-20 (Liu et al. 2020) – Clonotype 1

```
                    C  A  R  D  L  F  Y  Y  G  M  D  V  W
                   TGTGCGAGAGATCTATTTTACTACGGTATGGACGTCTGG
Germline sequence: TGTGCGAGAGA CTATT TACTACGGTATGGACGTCTGG
                     IGHV3-53 IGHD2-21        IGHJ6
```

b

CC12.1 (Rogers et al. 2020) – Clonotype 2

```
                    C  A  R  D  F  G  D  F  Y  F  D  Y  W
                   TGTGCGAGAGACTTCGGTGACTTCTACTTTGACTACTGG
Germline sequence: TGTGCGAGAGA   CGGTGACT CTACTTTGACTACTGG
                     IGHV3-53    IGHD4-17       IGHJ4
```

COVD21_P1_HC_A10-p1369 (Robbiani et al. 2020) – Clonotype 2

```
                    C  A  R  D  Y  G  D  F  Y  F  D  Y  W
                   TGTGCGAGGGATTACGGTGACTTCTACTTTGACTACTGG
Germline sequence: TGTGCGAGAGA TACGGTGACT CTACTTTGACTACTGG
                     IGHV3-53   IGHD4-17        IGHJ4
```

HbnC3t1p2_B10 (Kreer et al. 2020) – Clonotype 2

```
                    C  A  R  D  Y  G  D  Y  F  F  D  Y  W
                   TGTGCGAGAGATTATGGTGACTACTTCTTTGACTACTGG
Germline sequence: TGTGCGAGAGA   TGGTGACTACTACTTTGACTACTGG
                     IGHV3-66    IGHD2-21       IGHJ4
```

Supplementary Figure 1. Putative germline sequences for CDR H3 in representative antibodies from clonotypes 1 and 2. Amino acid and nucleotide sequences of the V-D-J junction are shown for **(a)** four clonotype 1 antibodies from different studies, and **(b)** three clonotype 2 antibodies from different studies. Putative germline sequences and segments are indicated. Somatically mutated nucleotides are underlined. Intervening spaces at the V-D and D-J junctions are N-nucleotide additions.

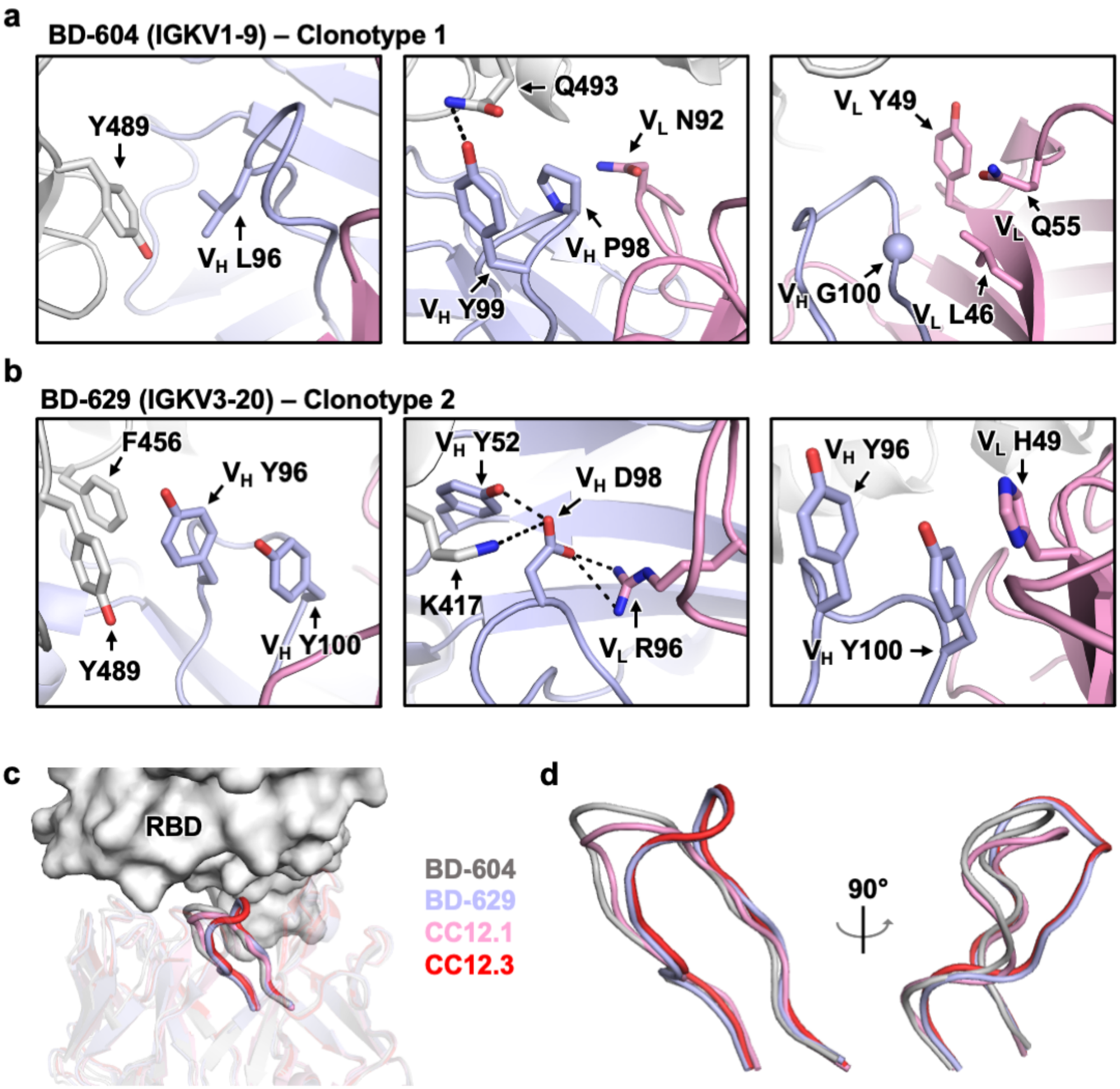

e

RMSD (Å) between CDR H3 loops

	BD-604	CC12.1	CC12.3
CC12.1	0.41		
CC12.3	1.5	1.14	
BD629	0.83	0.77	0.27

f

IGKV1-9	BD-604:	DLGPYGMDV
	CC12.1:	DLDVYGLDV
IGKV3-20	CC12.3:	DFGDFYFDY
	BD-629:	DYGDYYFDY
		95 96 97 98 99 100 100a 101 102

Supplementary Figure 2. Interactions between CDR H3 and the light chain of two clonotypes. (a) Interactions of L96, P98 and G100 (Kabat numbering) in CDR H3 of BD-604 (PDB 7CH4) with the IGKV1-9 light chain and SARS-CoV-2 RBD. **(b)** Interactions of Y96, D98 and Y100 (Kabat numbering) in CDR H3 of BD-629 (PDB 7CH5) the IGKV3-20 light chain and SARS-CoV-2 RBD. Gray: RBD; light blue: heavy chain; pink: light chain. V_H and V_L indicate residues belong to the heavy and light chain of the antibody, respectively. **(c-d)** Structural alignment of four antibodies highlighting the structural similarities and differences of the CDR H3 loop. Gray: BD-604; light blue: BD-629; pink: CC12.1; red: CC12.3. **(c)** An overall view with RBD as white surface and antibodies as cartoon representations. The antibodies are semi-transparent except for the CDR H3 loops. **(d)** Zoomed-in views of the CDR H3 loops. **(e)** Root-mean-square deviation (RMSD) are shown for the atomic positions of the CDR H3 loops from different antibodies. **(f)** Sequences of CDR H3 loops from different antibodies are shown. Amino acids are labeled according to Kabat numbering.

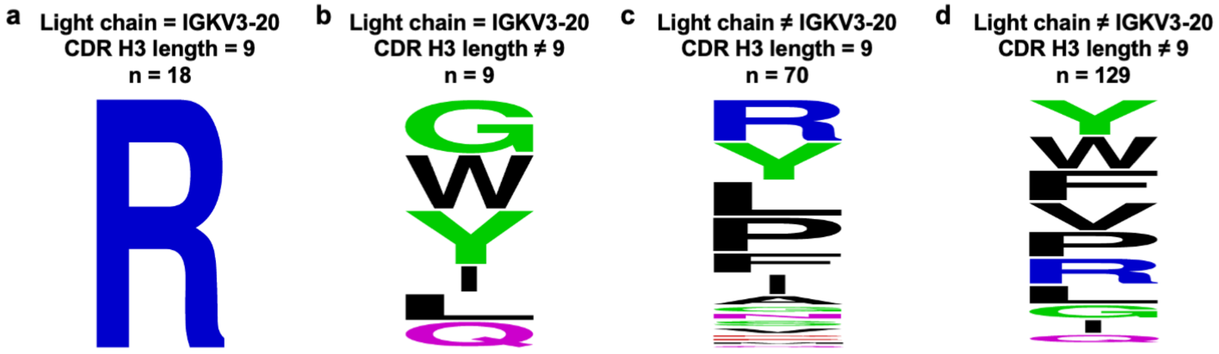

Supplementary Figure 3. Sequence logos of residue 96 in CDR L3 of IGHV3-53/3-66 RBD antibodies. (a) IGKV3-20 as light chain and CDR H3 length of 9 (n = 18), **(b)** IGKV3-20 as light chain and CDR H3 length not 9 (n = 9), **(c)** non-IGKV3-20 as light chain and CDR H3 length of 9 (n = 70), and **(d)** non-IGKV3-20 as light chain and CDR H3 length not equal to 9 (n = 129).

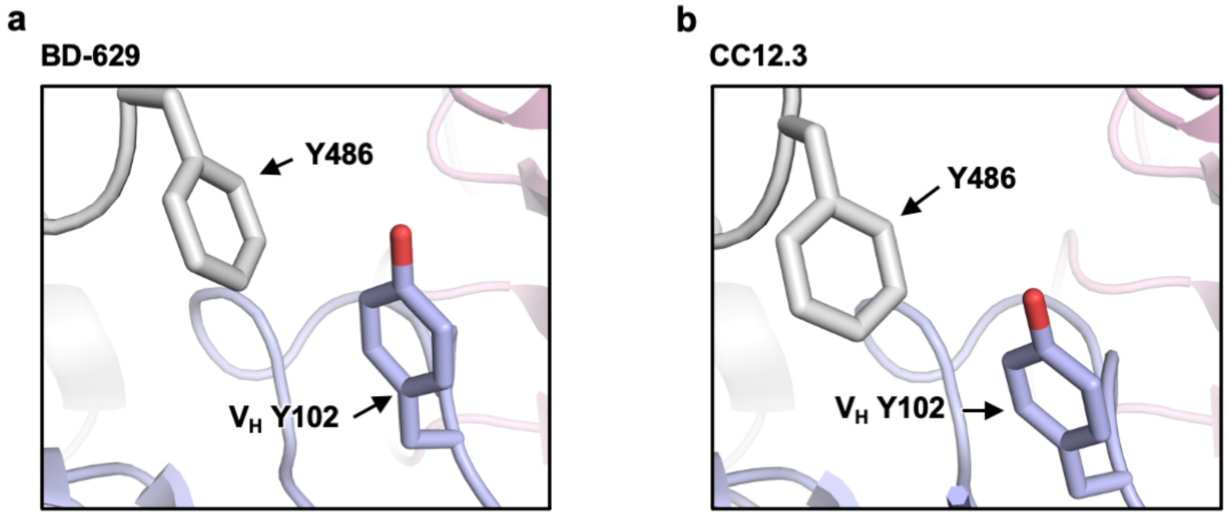

Supplementary Figure 4. Interactions between Y102 of CDR H3 loop of class 2 antibodies and the RBD of the S protein. (a) Interaction of Y102 (Kabat numbering) in CDR H3 of BD-629 (PDB 7CH5) with the RBD of the S protein. **(b)** Interaction of Y102 (Kabat numbering) in CDR H3 of CC12.3 (PDB 6XC4) with the RBD of the S protein. V_H indicates residues belong to the heavy chain of the antibody.

CC12.1 – Clonotype 1

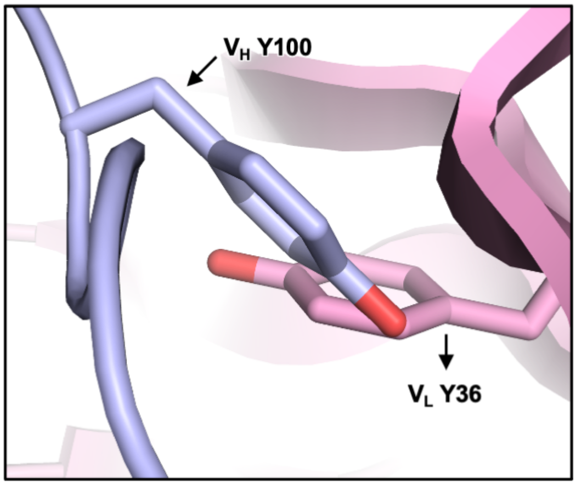

Supplementary Figure 5. V_H Y100 would clash with the light chain. Rosetta modeling[1] of G100 of CC12.1 (PDB 6XC2), a clonotype 1 antibody, to Y100 with a fixed backbone indicates a clash of V_H Y100 with V_L Y36. V_H and V_L indicate residues belong to the heavy and light chain of the antibody, respectively.

a

```
                                                    CDR H1                      CDR H2
COV107-23:  EVQLVESGGGLIQPGGSLRLSCAASGFTVSSNYMSWVRQAPGKGLEWVSVIYSGGSTFYADSVKGRFTI
COVD21-C8:  EVQLVESGGGLIQPGGSLRLSCAASGFTVSSNYMSWVRQAPGKGLEWVSVIYSGGSTFYADSVKGRFTI

                                                    CDR H3
COV107-23:  SRDNSKNTLYLQMNSLRAEDTAVYYCARDLGTGLFDYWGQGTLVTVSS
COVD21-C8:  SRDNSKNTLYLQMNSLRAEDTAVYYCARDWGDYYFDYWGQGTLVTVSS
                                         95 96 97 98 99 100 100a 101 102
```

b

COV107-23 (Robbiani et al. 2020) – Clonotype 1

```
                     C  A  R  D  L  G  T  G  L  F  D  Y  W
                    TGTGCGAGAGACCTCGGAACGGGGTTATTCGACTACTGG
Germline sequence:  TGTGCGAGAGA              GGTTATT GACTACTGG
                      IGHV3-53              IGHD3-22   IGHJ4
```

COVD21-C8 (Robbiani et al. 2020) – Clonotype 2

```
                     C  A  R  D  W  G  D  Y  Y  F  D  Y  W
                    TGTGCGAGAGACTGGGGAGACTACTACTTTGACTACTGG
Germline sequence:  TGTGCGAGAGACTGGGGA    ACTACTTTGACTACTGG
                      IGHV3-53  IGHD3-16          IGHJ4
```

Supplementary Figure 6. Putative germline sequences for CDR H3 in representative antibodies from clonotypes 1 and 2. (a) The heavy chains of COV107-23 (clonotype 1) and COVD21-C8 (clonotype 2) RBD antibodies from Robbiani et al.[2] are aligned as shown. CDRs are annotated based on Kabat numbering. Residues in COV107-23 that differ from COVD21-C8 are highlighted in yellow. **(b)** Amino acid and nucleotide sequences of the V-D-J junction of COV107-23 and COVD21-C8 are shown. Putative germline sequences and segments are indicated. COV107-23 and COVD21-C8 are named as COV107_Plate2_HC_23-P1369 and COVD21_P1_HC_C8-p1369, respectively, in Supplementary Table 1 of Robbiani et al.[2]

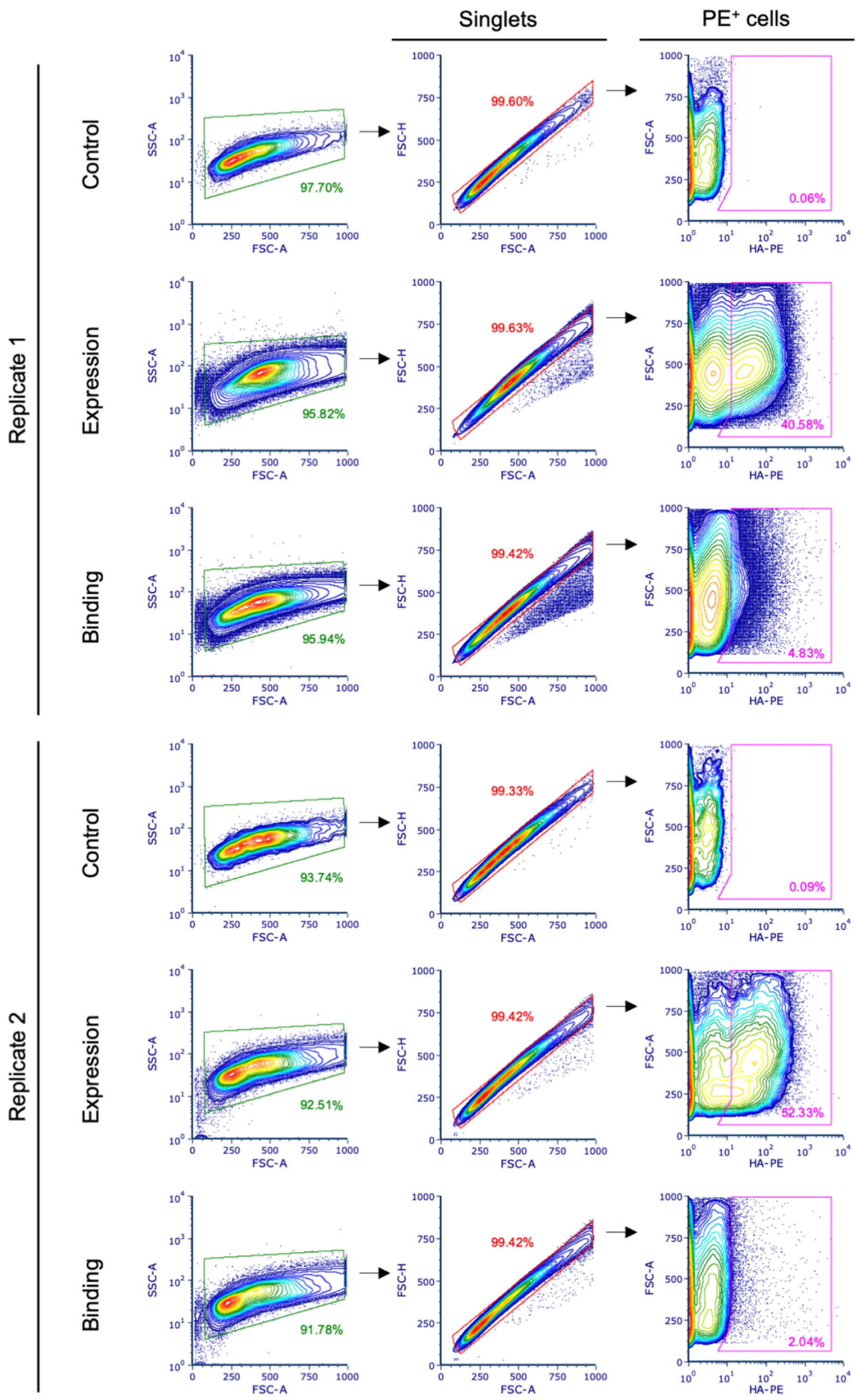

Supplementary Figure 7. Density plots of fluorescence-activated cell sorting of yeast cells. Cells from the B38 antibody yeast display library were sorted based on signal of PE. PE-positive singlets were collected.

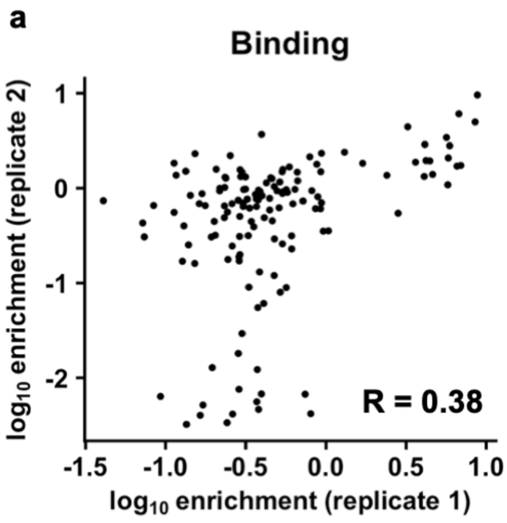

Supplementary Figure 8. Correlation between biological replicates of the yeast display selection experiment for 143 CDR H3 variants in the B38 antibody. (a) Correlation of log_{10} enrichment in binding between biological replicates is shown. **(b)** Correlation of log_{10} enrichment in expression between biological replicates is shown. The Pearson correlation (R) is indicated. Enrichment in binding and expression data are provided as Supplementary Data 2.

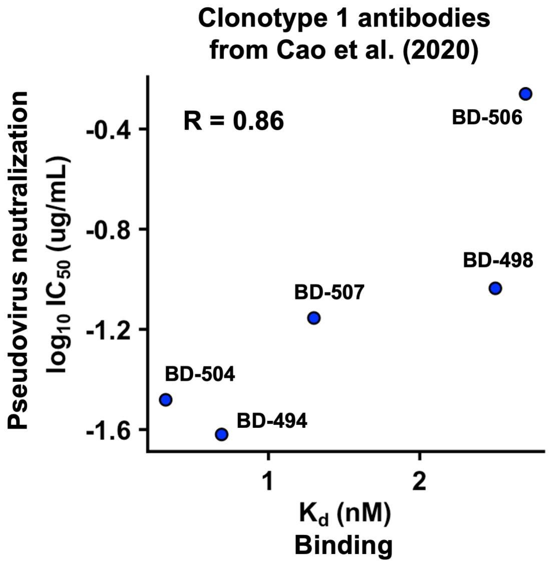

Supplementary Figure 9. Correlation between binding affinity and neutralization potency of clonotype 1 antibodies. Correlation between pseudovirus neutralization potency and binding affinity of five clonotype 1 antibodies (IGHV3-53/3-66 + IGKV1-9 with 9-residue CDR H3) from Cao et al.[3] is shown, with a Pearson correlation coefficient (R) of 0.86.

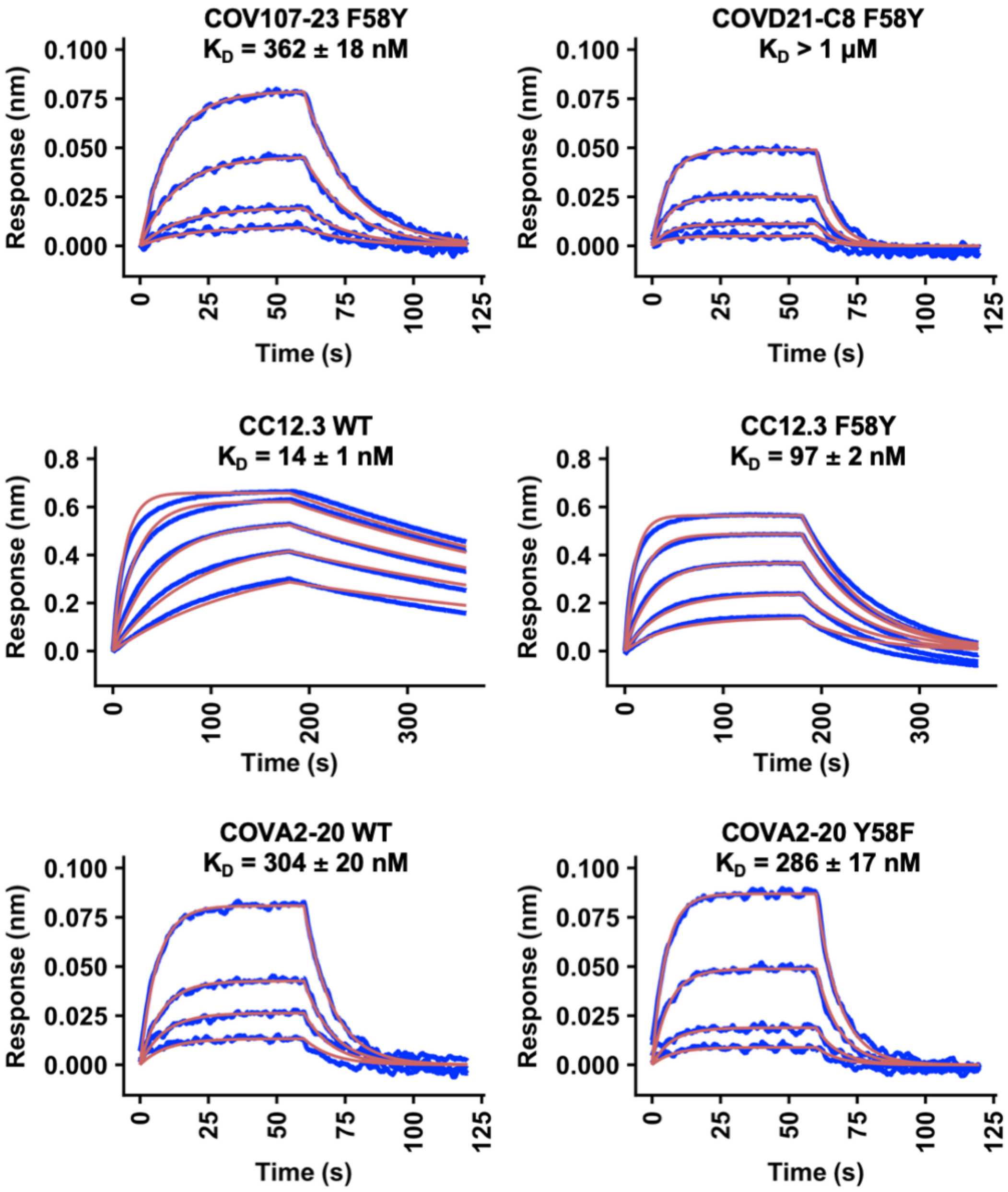

Supplementary Figure 10. Sensorgrams for binding of Fabs to SARS-CoV-2 RBD. Binding kinetics of different Fabs against recombinant SARS-CoV-2 RBD were measured by biolayer interferometry (BLI). Y-axis represents the response. Blue lines represent the response curve and red lines represent a 1:1 binding model. Binding kinetics were measured for four to five Fab concentrations.

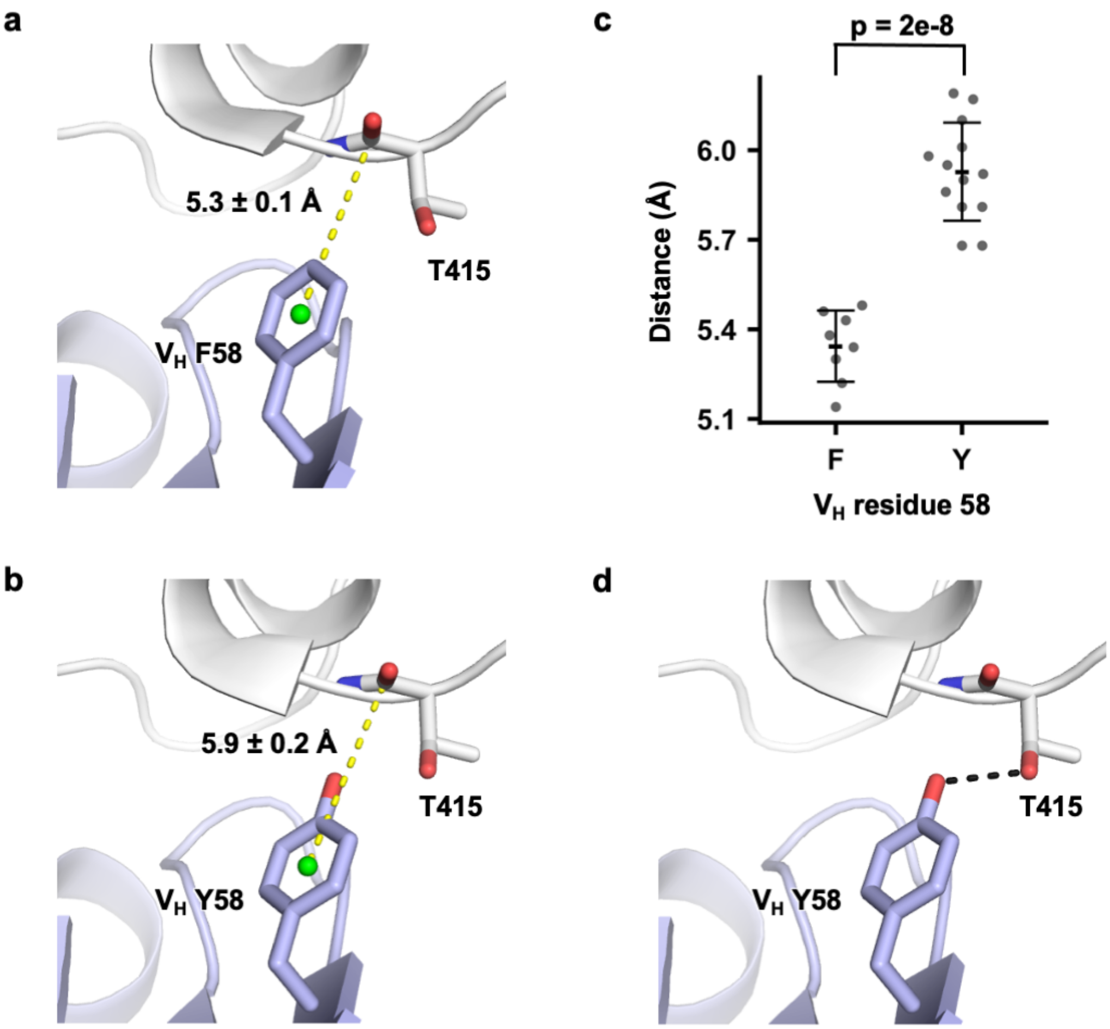

Supplementary Figure 11. Structural analysis of the Y58F mutation. (a) The distance between the centroid of the aromatic ring of V_H F58 and the backbone carbon atom of RBD T415 was measured for seven IGHV3-53/66 RBD antibodies with the Y58F mutation, namely P4A1 (PDB 7CJF), CC12.1 (PDB 6XC3), CC12.3 (PDB 6XC4), C102 (PDB 7K8M), BD-604 (PDB 7CH4), COVA2-04 (PDB 7JMO), and CB6 (PDB 7C01) (n.b., there are two copies of CC12.3-RBD complex in PDB 6XC4 and both copies were analyzed). The mean and standard deviation are indicated. The centroid of the aromatic ring is represented by the green sphere. Nucleotide sequences are publicly available for three antibodies with Y58F, namely CC12.1, CC12.3 and CB6[4,5]. For these three antibodies, Y58F occurs through a single point mutation in the second nucleotide of the codon as shown by IgBLAST analysis[6]. Specifically, the germline IGHV3-53/3-

66 gene contains TAC, which encodes Y58, while the antibodies CC12.1, CC12.3 and CB6 contain TTC, which encodes F58. **(b)** Same as panel **a**, except for nine IGHV3-53/3-66 RBD antibodies without a Y58F mutation, namely CV30 (PDB 6XE1), STE90-C11 (PDB 7B3O), B38 (PDB 7BZ5), P2C-1F11 (PDB 7CDI), C1A-B3 (PDB 7KFW), BD-629 (PDB 7CH5), C1A-F10 (PDB 7KFY), C1A-B12 (PDB 7KFV), and C1A-C2 (PDB 7KFX), (n.b., there are three copies of C1A-B3-RBD complex in PDB 7KFW and three copies of C1A-B12-RBD complex in PDB 7KFV, all of which were analyzed). **(c)** The distance data reported in panels **a** and **b** are shown. n = 8 distances were analyzed for F58 and n = 13 distances were analyzed for Y58. Error bars represent standard deviations. P-value was computed by two-tailed t-test with no adjustment for multiple comparisons. Source data are provided in the Source Data file. **(d)** The hydroxyl group of V_H Y58 can form an H-bond (black dashed line) with RBD T415. The hydrogen bond was identified by PISA (https://www.ebi.ac.uk/pdbe/pisa/)[7]. V_H indicates residues belong to the heavy chain of the antibody, respectively.

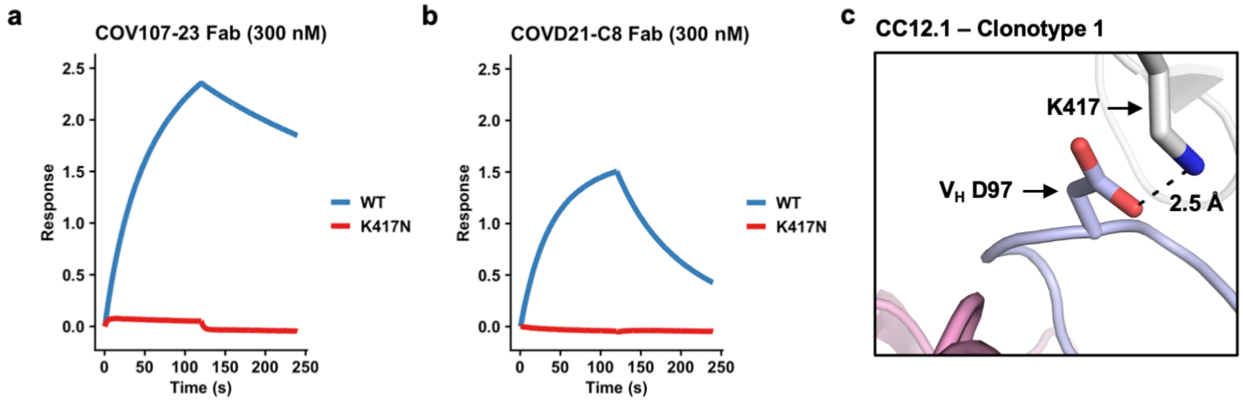

Supplementary Figure 12. Clonotypes 1 and 2 bind strongly to wild-type SARS-CoV-2 RBD but not to SARS-CoV-2 K417N RBD mutant. Binding kinetics of **(a)** COV107-23 (clonotype 1) and **(b)** COVD21-C8 (clonotype 2) Fabs to recombinant SARS-CoV-2 RBD were measured by biolayer interferometry. Y-axis represents the response. Blue and red lines represent the response curve for binding of Fabs to wild-type (WT) and K417N RBD mutant, respectively. **(c)** Electrostatic interaction between V_H D97 of CC12.1 (PDB 6XC2), a clonotype 1 antibody, with WT K417 RBD. V_H indicates residues belong to the heavy chain of the antibody.

Supplementary Tables

Supplementary Table 1. X-ray data collection and refinement statistics.

**Data collection**		
	COVA107-23 WT	COVA107-23 HC + COVD21-C8 LC
Beamline	SSRL 12-1	SSRL 12-1
Wavelength (Å)	0.97946	0.97946
Space group	P 2	P 1
Unit cell parameters		
a, b, c (Å)	79.4, 74.8, 172.7	73.3, 78.4, 91.6
α, β, γ (°)	90, 99.4, 90	100.1, 112.1, 92.8
Resolution (Å) [a]	50.0–1.98 (2.01–	50.0–3.30 (3.40–3.30)
Unique reflections [a]	130,795 (12,808)	24,557 (2,165)
Redundancy [a]	3.5 (2.9)	1.7 (1.6)
Completeness (%) [a]	94.3 (94.5)	82.3 (70.7)
$<I/\sigma_I>$ [a]	13.8 (1.0)	2.7 (1.0)
R_{sym}[b] (%) [a]	14.0 (>100)	29.9 (70.9)
R_{pim}[b] (%) [a]	5.7 (64.0)	18.8 (46.4)
$CC_{1/2}$[c] (%) [a]	99.7 (66.3)	93.4 (72.7)
**Refinement statistics**		
Resolution (Å)	30.7–1.98	35.9–3.30
Reflections (work)	130,416	24,493
Reflections (test)	6,632	1,186
R_{cryst}[d] / R_{free}[e] (%)	21.4/26.3	29.8/31.9
No. of atoms	14,029	12,881
Macromolecules	12,888	12,881
Solvent	1,137	0
Average *B*-value ($Å^2$)	38	59
Macromolecules	38	59
Solvent	41	N/A
Wilson *B*-value ($Å^2$)	30	54
**RMSD from ideal geometry**		
Bond length (Å)	0.012	0.014
Bond angle (°)	1.29	0.74
**Ramachandran statistics (%)**		
Favored	95.5	95.2
Outliers	0.06	0.00
**PDB code**	7LKA	7LK9

[a] Numbers in parentheses refer to the highest resolution shell.

[b] $R_{sym} = \Sigma_{hkl} \Sigma_i | I_{hkl,i} - <I_{hkl}> | / \Sigma_{hkl} \Sigma_i I_{hkl,i}$ and $R_{pim} = \Sigma_{hkl} (1/(n-1))^{1/2} \Sigma_i | I_{hkl,i} - <I_{hkl}> | / \Sigma_{hkl} \Sigma_i I_{hkl,i}$, where $I_{hkl,i}$ is the scaled intensity of the ith measurement of reflection h, k, l, $<I_{hkl}>$ is the average intensity for that reflection, and *n* is the redundancy.

[c] $CC_{1/2}$ = Pearson correlation coefficient between two random half datasets.

[d] $R_{cryst} = \Sigma_{hkl} | F_o - F_c | / \Sigma_{hkl} | F_o |$ x 100, where F_o and F_c are the observed and calculated structure factors, respectively.

[e] R_{free} was calculated as for R_{cryst}, but on a test set comprising 5% of the data excluded from refinement.

Supplementary Table 2. List of primers used in this study.

Primer Name	Sequence (5´ to 3´)
Oligo-F	ACC TAC AGA TGA ATT CTC TTA GGG CAG AAG ATA CCG CCG TCT ACT ACT GC
Oligo-R	GGG CCT TTT GTA GAA GCT GAA CTC ACA GTG ACG GTA GTC CCT TGT CCC CA
B38-VF	TGG GGA CAA GGG ACT ACC GTC ACT GTG
B38-VR	GCA GTA GTA GAC GGC GGT ATC TTC TGC
CDRH3-F	ACC TAC AGA TGA ATT CTC TTA GG
CDRH3-R	GGG CCT TTT GTA GAA GCT GAA CT

Supplementary References

1. Kellogg, E. H., Leaver-Fay, A. & Baker, D. Role of conformational sampling in computing mutation-induced changes in protein structure and stability. *Proteins: Struct. Funct. Bioinform.* **79**, 830-838 (2011).

2. Robbiani, D. F. *et al.* Convergent antibody responses to SARS-CoV-2 in convalescent individuals. *Nature* **584**, 437-442 (2020).

3. Cao, Y. *et al.* Potent neutralizing antibodies against SARS-CoV-2 identified by high-throughput single-cell sequencing of convalescent patients' B cells. *Cell* **182**, 73-84 (2020).

4. Yuan, M. *et al.* Structural basis of a shared antibody response to SARS-CoV-2. *Science* **369**, 1119-1123 (2020).

5. Shi, R. *et al.* A human neutralizing antibody targets the receptor-binding site of SARS-CoV-2. *Nature* **584**, 120-124 (2020).

6. Ye, J., Ma, N., Madden, T. L. & Ostell, J. M. IgBLAST: an immunoglobulin variable domain sequence analysis tool. *Nucleic Acids Res.* **41**, W34-W40 (2013).

7. Krissinel, E. & Henrick, K. Inference of macromolecular assemblies from crystalline state. *J. Mol. Biol.* **372**, 774-797 (2007).
