# Supplementary material for: Sequence signatures of two public antibody clonotypes that bind SARS-CoV-2 receptor binding domain
Source: Nat Commun. 2021 Jun 21;12:3815. doi: 10.1038/s41467-021-24123-7 (PMC8217500; doi:10.1038/s41467-021-24123-7)
Supplement: Supplementary file 3 — Description of Additional Supplementary Files [file 41467_2021_24123_MOESM3_ESM.pdf]

Description of Additional Supplementary Files

File Name: Supplementary Data 1
Description: List of IGHV3-53/3-66 RBD antibodies

File Name: Supplementary Data 2
Description: Counts, enrichment in expression and enrichment in binding data from deep sequencing of sorted cells

File Name: Supplementary Data 3
Description: List of 143 oligonucleotides for the construction of the B38 antibody yeast display library
