# Supplementary material for: Sequence signatures of two public antibody clonotypes that bind SARS-CoV-2 receptor binding domain
Source: Nat Commun. 2021 Jun 21;12:3815. doi: 10.1038/s41467-021-24123-7 (PMC8217500; doi:10.1038/s41467-021-24123-7)
Supplement: Supplementary file 7 — Reporting Summary [file 41467_2021_24123_MOESM7_ESM.pdf]

nature research

Corresponding author(s): Nicholas Wu

Last updated by author(s): Apr 24, 2021

Reporting Summary

Nature Research wishes to improve the reproducibility of the work that we publish. This form provides structure for consistency and transparency in reporting. For further information on Nature Research policies, see our Editorial Policies and the Editorial Policy Checklist.

Statistics

For all statistical analyses, confirm that the following items are present in the figure legend, table legend, main text, or Methods section.

n/a	Confirmed	
☐	☒	The exact sample size (n) for each experimental group/condition, given as a discrete number and unit of measurement
☐	☒	A statement on whether measurements were taken from distinct samples or whether the same sample was measured repeatedly
☐	☒	The statistical test(s) used AND whether they are one- or two-sided<br>*Only common tests should be described solely by name; describe more complex techniques in the Methods section.*
☒	☐	A description of all covariates tested
☒	☐	A description of any assumptions or corrections, such as tests of normality and adjustment for multiple comparisons
☐	☒	A full description of the statistical parameters including central tendency (e.g. means) or other basic estimates (e.g. regression coefficient) AND variation (e.g. standard deviation) or associated estimates of uncertainty (e.g. confidence intervals)
☐	☒	For null hypothesis testing, the test statistic (e.g. F, t, r) with confidence intervals, effect sizes, degrees of freedom and P value noted<br>*Give P values as exact values whenever suitable.*
☒	☐	For Bayesian analysis, information on the choice of priors and Markov chain Monte Carlo settings
☒	☐	For hierarchical and complex designs, identification of the appropriate level for tests and full reporting of outcomes
☐	☒	Estimates of effect sizes (e.g. Cohen's d, Pearson's r), indicating how they were calculated

Our web collection on statistics for biologists contains articles on many of the points above.

Software and code

Policy information about availability of computer code

Data collection	FACS Diva v8.0.1 software (BD Biosciences) was used for flow cytometry data collection.
Data analysis	Rosetta Commons v3.12 was used for in silico mutagenesis. FCS Express 6 software (De Novo Software) was used to analyze flow cytometry data. The links https://github.com/wchnicholas/IGHV3-53_sequence_features and https://github.com/timothyjtan/ighv3-53_3-66_antibody_sequence_features contain custom code for analysis.<br>HKL2000 (version 712) was used to process diffraction data. PHASER (version 2.1.2) was used to solve structures using molecular replacement. COOT (version 0.8.9) was used for iterative model building and PHENIX (version 1.12-2829) was used for refinement.

For manuscripts utilizing custom algorithms or software that are central to the research but not yet described in published literature, software must be made available to editors and reviewers. We strongly encourage code deposition in a community repository (e.g. GitHub). See the Nature Research guidelines for submitting code & software for further information.

Data

Policy information about availability of data

All manuscripts must include a data availability statement. This statement should provide the following information, where applicable:

- Accession codes, unique identifiers, or web links for publicly available datasets
- A list of figures that have associated raw data
- A description of any restrictions on data availability

Raw sequencing data have been submitted to the NIH Short Read Archive under accession number: BioProject PRJNA691562 [https://www.ncbi.nlm.nih.gov/bioproject/?term=PRJNA691562]. The X-ray coordinates and structure factors have been deposited to the RCSB Protein Data Bank under accession codes: 7LK9 [http://doi.org/10.2210/pdb7LK9/pdb] and 7LKA [http://doi.org/10.2210/pdb7LKA/pdb]. Source data are provided with this paper. Data for literature mining, biolayer interferometry, and deep sequencing analysis are available at https://github.com/wchnicholas/IGHV3-53_sequence_features,

the Supplementary Data and the Source Data files. Biological materials including the wild-type B38 yeast display plasmid, pCTcon2_B38, and the B38 yeast antibody display library are available by contacting Nicholas C. Wu. (nicwu@illinois.edu). All relevant data are available from the authors.

Field-specific reporting

Please select the one below that is the best fit for your research. If you are not sure, read the appropriate sections before making your selection.

- [x] Life sciences
- [ ] Behavioural & social sciences
- [ ] Ecological, evolutionary & environmental sciences

For a reference copy of the document with all sections, see nature.com/documents/nr-reporting-summary-flat.pdf

Life sciences study design

All studies must disclose on these points even when the disclosure is negative.

Sample size	Biological replicates for the yeast display screening were performed to assess reproducibility based on the correlation between replicates.
Data exclusions	No data was excluded from any analysis.
Replication	Replicated experiments are indicated in the figure legends.
Randomization	Not relevant to this study.
Blinding	Not relevant to this study.

Reporting for specific materials, systems and methods

We require information from authors about some types of materials, experimental systems and methods used in many studies. Here, indicate whether each material, system or method listed is relevant to your study. If you are not sure if a list item applies to your research, read the appropriate section before selecting a response.

Materials & experimental systems

n/a	Involved in the study	
[ ]	[x]	Antibodies
[ ]	[x]	Eukaryotic cell lines
[x]	[ ]	Palaeontology and archaeology
[x]	[ ]	Animals and other organisms
[x]	[ ]	Human research participants
[x]	[ ]	Clinical data
[x]	[ ]	Dual use research of concern

Methods

n/a	Involved in the study	
[x]	[ ]	ChIP-seq
[ ]	[x]	Flow cytometry
[x]	[ ]	MRI-based neuroimaging

Antibodies

Antibodies used	PE anti-HA.11, BioLegend, Clone 16B12, Cat. No. 901517,<br>PE anti-human IgG Fc, BioLegend, Clone HP6017, Cat. No. 409304
Validation	PE anti-HA.11 was validated via flow cytometry using CHO cells according to the manufacturer's webpage: https://www.biolegend.com/en-us/products/pe-anti-ha-11-epitope-tag-antibody-13535?GroupID=GROUP26.<br>PE anti-human IgG Fc was validated via flow cytometry on CHO cells according to the manufacturer's webpage: https://www.biolegend.com/en-us/products/pe-anti-human-igg-fc-7130?GroupID=GROUP23.

Eukaryotic cell lines

Policy information about cell lines

Cell line source(s)	EBY100 yeast cells were purchased from American Type Culture Collection. Expi293F cells were purchased from Thermo Fisher Scientific.
Authentication	EBY100 yeast cells and Expi293F cells were not authenticated.
Mycoplasma contamination	EBY100 yeast cells and Expi293F cells were not tested for mycoplasma contamination.
Commonly misidentified lines<br>(See ICLAC register)	No commonly misidentified cell lines were used in the study.

Flow Cytometry

Plots

Confirm that:

☒ The axis labels state the marker and fluorochrome used (e.g. CD4-FITC).

☒ The axis scales are clearly visible. Include numbers along axes only for bottom left plot of group (a 'group' is an analysis of identical markers).

☒ All plots are contour plots with outliers or pseudocolor plots.

☒ A numerical value for number of cells or percentage (with statistics) is provided.

Methodology

Sample preparation	EBY100 yeast cells were incubated with 1 ug/ml PE anti-HA.11 overnight at 4C for expression assay. For binding assay, EBY100 yeast cells were incubated with 20 ug/ml SARS-CoV-2 RBD-IgG Fc overnight at 4C, then with 1 ug/ml PE anti-human IgG Fc for 1 h at 4C.
Instrument	BD FACSAria II Cell Sorter
Software	Flow cytometry data was collected with BD FACS Diva software and analyzed with FCS Express 6 software.
Cell population abundance	Abundance of the relevant cell populations collected are shown in Supplementary Figure 7.
Gating strategy	Singlets were gated from the starting cell population. Then PE positive cells were gated from singlets.

☒ Tick this box to confirm that a figure exemplifying the gating strategy is provided in the Supplementary Information.
